# Antibody formation to enzyme therapy in classic infantile Pompe disease: implications of patient age

**DOI:** 10.1186/1471-2474-14-S2-P18

**Published:** 2013-05-29

**Authors:** C van Gelder, M Kroos, L Özkan, I Plug, A Reuser, A van der Ploeg

**Affiliations:** 1 Department of Pediatrics, Center for Lysosomal and Metabolic Diseases, Erasmus MC University Medical Center, Rotterdam, The Netherlands; 2 Department of Clinical Genetics, Center for Lysosomal and Metabolic Diseases, Erasmus MC University Medical Center, Rotterdam, The Netherlands

## Introduction

Enzyme-replacement therapy (ERT) with alglucosidase alfa has improved the lifespan of patients with classic infantile Pompe disease, although ERT is not effective in a subset of patients who mount an immune response to the exogenous enzyme. We studied the development of antibodies in response to ERT and its effect on clinical outcomes in 11 patients with classic infantile Pompe disease treated with ERT since 1999 for a median of 4.2 years (range 3 months to 12 years).

## Results

We determined the endogenous acid alpha-glucosidase expression (Cross-Reactive Immunological Material, or CRIM status) and regularly assessed antibody formation in all 11 patients, all of whom developed antibodies. The patients who lacked any endogenous acid alpha-glucosidase production (CRIM-negative, n=3) did not develop a substantially different antibody titer than those who produced an inactive form of the enzyme (n=8). However, the patient’s age at the start of ERT proved to be important: none of the four patients who started ERT before 2 months of age developed titers of more than 1:6,250. Gross motor function and cardiac dimension improved less in patients with high titers and in CRIM-negative patients. The three CRIM-negative patients have died, whereas the eight CRIM-positive patients survived.

## Conclusion

Antibody formation is common. High antibody titers and a CRIM-negative status are associated with a poorer clinical outcome. Earlier initiation of ERT may prevent the formation of a severe immune response.

